# The Weight of a Like on Social Networks: How Self-Monitoring Moderates the Effect of Cyber-Ostracism

**DOI:** 10.5334/irsp.855

**Published:** 2024-05-06

**Authors:** Alessandra Sacino, Antonio Aquino, Daniele Paolini, Luca Andrighetto

**Affiliations:** 1 Department of Neuroscience, Imaging, and Clinical Sciences, Chieti-Pescara University, Chieti, IT; 2 Department of Health Sciences, Magna Graecia University of Catanzaro, Catanzaro, IT; 3 Faculty of Human Sciences, Education and Sport, Pegaso University, Naples, IT; 4 Department of Education, University of Genova, Genoa, IT

**Keywords:** Ostracism, Cyber-ostracism, Social exclusion, Social-media, Self-Monitoring

## Abstract

Cyber-ostracism is an experience that, similar to the ostracism occurring within in-person relational contexts, gives rise to negative psychological consequences, leading to negative emotional reactions, and threatening the basic needs of each individual-belonging, self-esteem, meaningful existence, and control. The present study aimed to explore the possible moderating role of self-monitoring on the impact of cyber-ostracism on people’s emotions and need satisfaction. We employed the Ostracism Online paradigm, a research tool resembling a social media platform, that allows researchers to manipulate the number of likes that participants receive as a cue of cyber-ostracism. A total of 212 participants were randomly assigned to one of two experimental conditions (Ostracism Online: cyber-ostracism vs. cyber-inclusion). After completing measures related to their social media usage and the self-monitoring scale, participants were exposed to the Ostracism Online paradigm and then were asked to complete measures related to their emotional reactions and need satisfaction. Results highlighted a different moderating role of self-monitoring on emotions and need satisfaction. Specifically, in the cyber-ostracism condition, participants with higher levels of self-monitoring reported higher levels of negative emotions compared to participants with lower levels of self-monitoring. Differently, we only found an effect of self-monitoring on the control dimension of need satisfaction. In particular, included participants with higher levels of self-monitoring reported higher levels of perceived control compared to included participants with lower levels of self-monitoring. Our findings contribute to expanding our understanding of self-monitoring and its role in moderating the effects of cyber-ostracism that may occur within social media.

## Introduction

The increased diffusion and availability of the internet and the new communication technologies among people (i.e., smartphones, personal computers, and social networks), enriched their social life by allowing them to interact with acquaintances and friends or make new ones (Riva, 2014; Timeo et al., 2020). However, there is also a dark side associated with internet usage, related to the abusive and unsafe use of electronic communication systems that may expose people to a new form of ostracism known as ‘cyber-ostracism’, i.e., being ignored and excluded during online social interactions (Williams et al., 2000). Cyber-ostracism is an experience that, similar to the ostracism occurring within in-person relational contexts, gives rise to negative psychological consequences, threatening the basic needs of each individual (need for belonging, self-esteem, meaningful existence and control; van Beest & Williams, 2006). One of the most used social networks across the world is Facebook, with 1.96 billion people having a Facebook account (Meta Platforms, Inc., 2022). Facebook allows people to create a personal profile and expand their social network by asking other users for friendship, mutually viewing their profiles, and commenting on their posts. With regard to the ostracism perpetuated on Facebook, some studies have recently suggested that the number of comments received by others may be considered a cue of cyber-ostracism. Indeed, people who do not receive any comments on their posts show a lower sense of belonging than those who receive at least one comment (Tobin et al., 2015). Furthermore, the ‘like’ button allows users to interact and evaluate others simply and immediately: Facebook users can like their friends’ posts and, also, receive likes from them.

Wolf et al. (2015) developed the Ostracism Online paradigm, a social media platform for cyber-ostracism research that simulates an ostracism experience in a social media context. In their research, Wolf et al. (2015) highlighted that people who do not receive any likes on their posts or profiles show a significant lowering of basic needs satisfaction, which is a typical consequence of ostracism. This confirms how a seemingly simple activity such as receiving likes may somewhat contribute to undermining people’s well-being. These findings have been recently replicated by Timeo et al. (2020) by considering a sample of preadolescents. Adopting the Ostracism Online paradigm, they showed that those who received fewer likes than others reported higher levels of need threat and negative emotions. However, social networks are also important for adults, as shown by the intensive use of Facebook and other social networks during the Covid-19 emergency (Jaspal et al., 2020). In fact, during that period, social networks represented for most people a way to virtually escape from home and stay connected with friends and acquaintances. Accordingly, the Ostracism Online task has also been employed in studies involving adult samples (e.g., Lutz & Schneider, 2021; Marinthe et al., 2022; Reich et al., 2023), revealing similar effects to those that emerge when considering younger samples. Thus, this paradigm has so far been shown to be ecologically valid in exploring people’s reactions to cyber-ostracism across a wide range of age groups. Instead, little is known about the possible factors that may moderate reactions to this form of ostracism, especially when compared to the vast literature that has studied moderating variables involved in reactions to other forms of ostracism.

Empirical literature clearly revealed that reactions to ostracism are modulated both by dispositional and contextual factors. With regard to dispositional factors, narcissism (Twenge, 2005; Twenge & Campbell, 2003) and rejection sensitivity (Ayduk et al., 2008) received greater attention. Narcissists aim to exhibit a positive social image of themselves and react negatively when they fail to pursue this purpose. Ostracism tends to elicit higher anger and aggressive reactions among narcissists than among non-narcissist participants, as it threatens their egotism (Twenge & Campbell, 2003). Similarly, previous literature has shown that people who are high on rejection sensitivity anxiously expect, readily perceive, and overreact to rejection (Downey & Feldman, 1996). Moreover, scholars showed that the consequences of being ostracized are affected by personal sexual orientation. Being ostracized, indeed – but not being included – lowered the working memory capacity of gay men relative to heterosexual men (Paolini et al., 2020). Moving to contextual factors, some studies showed that group membership may play a moderating role (e.g., Krill & Piatek, 2009; Paolini et al., 2016), given that being ostracized from an ingroup member can lead to more intense physiological reactions rather than being ostracized from an outgroup member. However, a lack of consistency in previous studies should be noted, as other studies showed that group membership does not affect the consequences of ostracism (e.g., Fayant et al., 2014; Jauch et al., 2022; Zadro et al., 2004).

In analyzing the moderating role of individual and contextual variables involved in reactions to ostracism, research has also stressed the importance of considering the time point at which these variables seem to intervene. According to the Temporal Need Threat Model (Williams, 2009), reactions to ostracism (i.e., an impact on fundamental need satisfaction) can occur immediately (reflexive stage) and at a delayed time point (reflective stage). Based on the assumption that the response to ostracism is mainly reflexive in nature, several studies (see Williams & Nida, 2022, for a review) revealed null or weak relations between moderating variables and pain or distress immediately felt after experiencing ostracism, while showing more consistent relations in the subsequent reflective stage. However, the meta-analysis by Hartgerink et al. (2015) documented that moderation can also be observed at a reflexive stage, and thus some moderating variables also intervene at this stage. Taken together, the evidence shown above clearly reveals that the knowledge of possible variables shaping people’s reactions to ‘classical’ forms of ostracism is quite consistent. Instead, so far little is known about possible factors modulating the effects of experiencing ostracism in social networks. In this sense, shedding light on possible individual factors that modulate people’s reactions to this emergent form of ostracism could be highly relevant, as they may be peculiar or have a nature different from those featured in other forms of ostracism.

To fill this gap, in the present research, we proposed for the first time self-monitoring (Snyder, 1974) as a crucial dispositional factor in moderating the impact of cyber-ostracism. The psychological construct of self-monitoring refers to the regulation of expressive and self-presentational behaviors in social situations. The self-monitoring theory proposes that individuals systematically vary in the extent to which they are willing and able to monitor and control their expressive behaviors and public appearances. Individuals known as high self-monitors are particularly aware of and responsive to social cues. The images that they present are variable and tailored to situational context. In contrast, low self-monitors value consistent behavior that reflects what they perceive to be their true selves. Low self-monitors are typically less reactive to social circumstances and possess smaller repertoires of self-presentational skills.

Considering the importance that people with higher levels of self-monitoring give to their social image, we reasoned that they could be more sensitive to those events that characterize online social interactions, such as the likes received by others, as a signal of social acceptance. Consequently, we hypothesized that self-monitoring would moderate the effect of cyber-ostracism on emotions (HP1) and need satisfaction (HP2). Because the literature on ostracism has shown that emotions and need satisfaction are distinct outcomes of ostracism (Buckley et al., 2004; Chow et al., 2008; see Eck & Riva, 2016, for a review), we explored the moderating role of self-monitoring by considering these variables as separate outcomes.

Specifically, regarding the emotions (HP1), we expected that cyber-ostracized participants (vs. included) with higher levels of self-monitoring (vs. participants with lower levels of self-monitoring) would express more negative emotions given that their social image did not fit with their desire to be socially accepted by others. Similarly, regarding need satisfaction (HP2), we expected that participants with higher levels of self-monitoring (vs. participants with lower levels of self-monitoring) would express a lower need satisfaction when they are cyber-ostracized compared to cyber-included participants.

## Methods

### Participants and Experimental Design

An a priori power analysis was conducted using G*Power 3.1 (Faul et al., 2007), adopting the “ANOVA: Fixed, special, main, interactions” method, revealing that at least 210 participants were required to observe a medium effect size (*f* = 0.25), with α = .05 and power = .95. Participants were recruited among students from two large Italian universities located in the North and Center of Italy. Students who did not possess a Facebook account were excluded from participation. Two hundred and twelve students participated in the study (158 females, *M_age_* = 21, *SD_age_* = 5.16) and were randomly assigned to one of two experimental conditions (Ostracism Online: cyber-ostracism vs. cyber-inclusion).

### Procedure

Students who agreed to participate in the study received a personal link on their e-mail address, redirecting them to Amazon’s Web Services platform, which hosted the entire experiment. The experiment was presented as a study regarding the opinions and habits of young people using social media. Participants were informed that they would be required to complete questionnaires about their habits, interact with other participants on an online platform, and evaluate their experience on the platform. After giving informed consent, participants completed a short survey regarding their social media usage. Then, they were asked to enter their demographics (i.e., age and gender) and complete the Self-Monitoring Scale (Snyder & Gangestad, 1986). Afterwards, they were exposed to the Ostracism Online paradigm (Wolf et al., 2015), and they were randomly assigned to one of the two experimental conditions (cyber-ostracism vs. cyber-inclusion). Participants were induced to think they would be connected with other people through the internet, but, instead, only one participant at a time was involved, whilst all other members were computerized, and all of their actions were simulated through computer scripts. Following the interaction, participants were asked to think about how they felt during the interaction activity and to complete the Need-Satisfaction Scale (Zadro et al., 2004; Williams et al., 2000), the Emotional Reaction Scale (Buckley et al., 2004), the manipulation and the attention check questions. The entire experiment required approximately 20 minutes. Eventually, participants were fully debriefed on the purposes of the study and the nature of the deception and asked again to provide informed consent.

### Ostracism Online

We adopted the paradigm developed by Wolf and colleagues (2015), adapted in Italian. Ostracism Online is a web-based ostracism task resembling a social media website that uses ‘likes’ to either socially include or exclude participants. At the beginning of the task, participants were asked to create a personal profile by choosing a nickname and writing a description to introduce themselves to other participants. After creating their profiles, participants were told that, during the upcoming three minutes, they would be able to virtually interact with the other participants. They could see their profiles, read their description and react to them using a ‘like’ button, similar to the like button on Facebook. Participants were therefore asked to click ‘continue’ when they were ready to proceed and interact with the other participants. Hence, they were presented with a webpage in which their profile was visible along with other eight profiles, supposedly created by other participants, that were, instead, pre-programmed scripts (four male and four female). On this page, participants were required to read the descriptions - translated and adapted in Italian from the original paradigm, try to form an impression of the others, and react to their profiles by clicking the ‘like’ button. Whenever they received a ‘like’ from one of the pre-programmed participants, a pop-up notification informed them, showing the name of the participant who liked their profile (e.g., ‘Sandro liked your profile’). The number of ‘likes’ received was displayed at the bottom of each profile, increasing with each new like received. Whilst participants were induced to think they were receiving ‘likes’ from the other participants, they were, in fact, randomly assigned to one of two experimental conditions and received a pre-programmed number of ‘likes’: six in the cyber-inclusion condition and one in the cyber-ostracism condition. With regard to the number of ‘likes’ received by the pre-programmed profiles, they ranged from four to seven, meaning that included participants received a number of ‘likes’ close to the average (*n* = 5). In contrast, cyber-ostracized participants received a number of ‘likes’ clearly below the average. Following the three-minute interaction, participants were shown a ranking table, displaying all ‘likes’ received by the participant along with the pre-programmed scripts, from the most liked profile to the least liked one.

### Measures

#### Self-Monitoring Scale

Before Ostracism Online, participants completed the Italian version (Delle Grazie, 2008) of the Self-Monitoring Scale (Snyder & Gangestad, 1986). The scale consists of 18 items – 10 items were reverse scored. Participants rated their agreement with the statements (e.g., ‘I guess I put on a show to impress or entertain people’, ‘I would probably make a good actor’) on a scale ranging from 1 (*totally disagree*) to 7 (*totally agree)*. Scores were then averaged to obtain a Self-Monitoring index (α = .84). Higher scores indicated a tendency to adequate their behaviors to the social context.

#### Need Satisfaction Scale

Participants were then asked to complete the Need-Satisfaction Scale (Zadro et al., 2004; Williams et al., 2000) consisting of 20 items. Participants were asked to rate, on a 7-point scale (*1 = not at all; 7 = completely*), the degree to which they experienced feelings of belongingness (e.g., ‘I felt excluded’, ‘The other participants interacted with me’, α = .85), self-esteem (e.g., ‘I felt liked’, ‘I felt satisfied’, α = .86), control (e.g., ‘I felt in control during the interaction with the other participants’, ‘I felt able to significantly modify the events’, α = .79), and meaningful existence (e.g., ‘I felt invisible’, ‘I felt useful’, α = .84) during the Ostracism Online administration. After reverse-coding negative items, we average responses both into a single score for each need and into an overall need satisfaction index (α = .90), with lower ratings indicating higher need threat and higher ratings indicating higher need satisfaction.

#### Rejected-Related Emotions Scale

Emotional reactions to the Ostracism Online experience were measured with the Rejected-Related Emotions Scale (Buckley et al., 2004), consisting of 15 items. Participants were asked to rate on a 7-point scale (*1 = not at all; 7 = completely*) the extent to which they experienced happiness (three items, α = .94), anger (three items, α = .95), hurt feelings (three items, α = .97), anxiety (three items, α = .89), and sadness (three items, α = .93) during the Ostracism Online administration. After reverse coding the happiness index, we averaged emotions responses into a single overall negative emotions index (α = .84), with higher ratings indicating higher negative emotion.

#### Attention check

Participants were asked to indicate, approximately, the number of likes they received during the interaction, on a scale from 1 to 5, where 1 = ‘less than two’, 2 = ‘three or four’, 3 = ‘five or six’, 4 = ‘more than 7’, 5 = ‘I don’t remember’.

#### Manipulation checks

To check for the effectiveness of cyber-ostracism manipulation, participants were asked to rate how much they felt excluded and ignored during the interaction task on a scale ranging from 1 (*not at all*) to 7 (*extremely*). We averaged the score across these two items (*r* = .89, *p* < .001).

## Data Analyses Overview

Analyses were conducted using the GAMLj package (Gallucci, 2019) in Jamovi 2.2 version (The Jamovi project, 2021). To test whether self-monitoring moderates the effect of online ostracism on emotions and need satisfaction, general linear models were run on our dependent variables (i.e., negative emotions and need satisfaction), including the experimental condition as the independent variable (cyber-ostracism = 1 vs. cyber-inclusion = 2)^1^ and self-monitoring as moderating variable.

### Results

#### Attention and Manipulation Checks

With regard to the attention check, only five participants were not able to correctly recall the number of likes they received during the interaction. These participants were, hence, excluded from the analyses.^2^

Moreover, we evaluated the effectiveness of the Ostracism Online manipulation. Cyber-ostracized participants felt more ignored (*M* = 5.19, *SD* = 1.32) and ostracized (*M* = 4.51, *SD* = 1.45) than cyber-included participants (*M* = 1.56, *SD* = 0.82; *M* = 2.11, *SD* = 0.35, respectively), *F*(1, 205) = 525, *p* < .001, partial *η^2^* = .72; *F*(1, 205) = 237, *p* < .001, partial *η^2^* = .54, respectively. Taken together, these findings confirmed the effectiveness of our manipulation.

#### Moderation of Self-monitoring on Rejected-related Emotions

To test our hypotheses that self-monitoring moderates the link between cyber-ostracism and our dependent variables, we run different moderation analyses.

**Figure 1:**
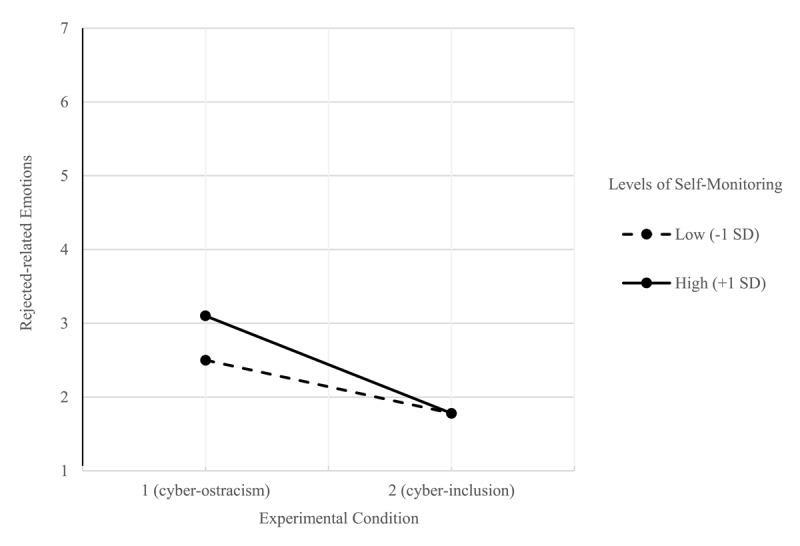
The interaction effect of cyber-ostracism and self-monitoring on perceived rejected-related emotions.

In the first model, we ran a moderation analysis including the experimental condition (cyber-ostracism = 1 vs. cyber-inclusion = 2) as the independent variable, self-monitoring as the moderator, and the overall negative emotions index as the outcome variable. Results indicated a main effect of the experimental condition, *F*(1, 203) = 52.52, *p* < .001, partial *η^2^* = .20. Overall, cyber-ostracized participants reported higher levels of negative emotions (*M* = 2.78, *SD* = 1.21) than cyber-included participants (*M* = 1.83, *SD* = .42). A main effect of self-monitoring also emerged as significant, *F*(1, 203) = 5.56, *p* = .019, partial *η^2^* = .03. Self-monitoring was positively associated with negative emotions, *b* = 0.16, 95% C.I. [0.02, 0.29]. Lastly, the two-way experimental condition × self-monitoring interaction emerged as significant, *F*(1, 203) = 4.19, *p* = .042, 95%C.I. [–0.55, –0.01], and was interpreted through a simple slope analyses. Both participants with lower levels (–1SD) and higher levels of self-monitoring (+1SD) reported higher levels of negative emotions in the cyber-ostracism condition (vs. cyber-inclusion). However, the magnitude of the effect was larger for participants with higher levels of self-monitoring. Consistent with HP1, participants with higher levels of self-monitoring (+1SD) reported higher levels of negative emotions in the cyber-ostracism condition, *b* = –1.22, *SE* = 0.18, 95%CI [–1.58, –0.85], *t*(203) = –6.58, *p* < .001, compared to participants with lower levels of self-monitoring (–1SD), *b* = –0.68, *SE* = 0.18, 95%CI [–1.05, –0.32], *t*(203) = –3.69, *p* < .001, (see Figure 1). Furthermore, confirming this pattern of findings, we also ran a simple slope analysis by comparing the effects of cyber-ostracism (vs. cyber-inclusion). For participants in the cyber-ostracism condition, self-monitoring was positively associated with negative emotions, *b* = 0.30, *SE* = 0.10, 95%CI [0.10, 0.5], *t*(203) = 3.027, *p* = 0.003. On the contrary, participants in the cyber-inclusion condition displayed similar levels of negative emotions regardless of their levels of self-monitoring, *b* = 0.02, *SE* = 0.09, 95%CI [–0.16, 0.21], *t*(203) = 0.227, *p* = 0.820.

#### Moderation of Self-monitoring on Need Satisfaction

We then ran a second model that included the experimental condition (cyber-ostracism = 1 vs. cyber-inclusion = 2) as the independent variable, self-monitoring as moderator, and need satisfaction as the outcome variable. Although the Temporal Need Threat Model proposes that ostracism equally threatens the four fundamental needs, belongingness, self-esteem, meaningful existence, and control (e.g., Williams, 2009), few studies that employed the Ostracism Online paradigm (e.g., Ren et al., 2021; Timeo et al., 2020) did not find direct effects of cyber-ostracism on the control dimension. Hence, we reasoned to perform analyses on both the need satisfaction overall index and each of the sub-components of the scale.

Starting from the overall need satisfaction index, only the effect of the experimental condition emerged as significant. Participants in the cyber-ostracism condition reported lower levels of need satisfaction (*M* = 3.21, *SD* = 1.05) than cyber-included participants (*M* = 4.92, *SD* = 0.79), *F*(1, 203) = 168.43, *p* < .001, partial *η^2^* = .45. The experimental condition × self-monitoring interaction, instead, did not emerge as significant, *F*(1, 203) = 1.35, *p* = .247, 95%C.I. [–0.11, 0.44].

**Figure 2:**
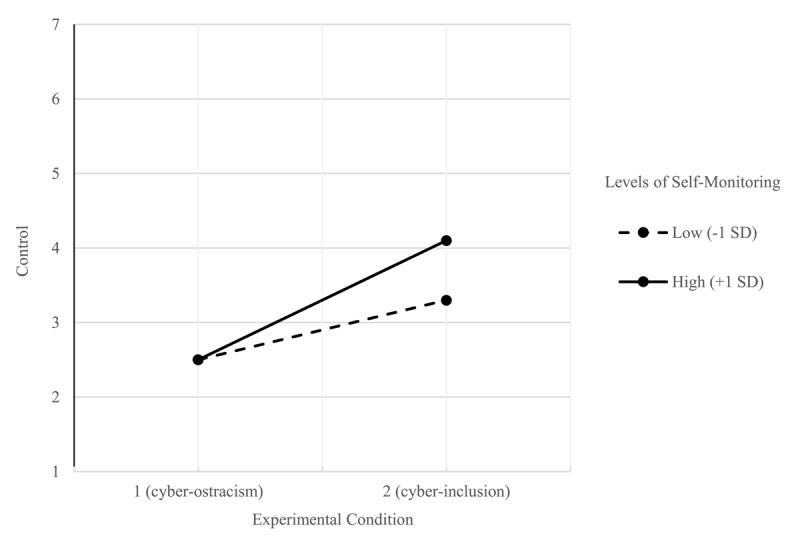
The interaction effect of cyber-ostracism and self-monitoring on perceived control.

With regard to the sub-components of need satisfaction, we found significant effects of self-monitoring only when considering the perceived sense of control as the outcome variable. In this GLM model, we included the experimental condition (cyber-ostracism = 1 vs. cyber-inclusion = 2) as the independent variable, self-monitoring as the moderator, and perceived control as the outcome variable. Results indicated a main effect of the experimental condition. Participants in the cyber-ostracism condition reported lower levels of control (*M* = 2.55; *SD* = 0.99) than participants in the cyber-inclusion condition (*M* = 3.69, *SD* = 1.35), *F*(1, 203) = 51.51, *p* < .001, partial *η^2^* = .20. Furthermore, results also indicated a main effect of self-monitoring, *F*(1, 203) = 4.91, *p* = .028, partial *η^2^* = .02. Self-monitoring was positively associated to perceived control, *b* = 0.19, 95% C.I. [0.02, 0.35]. The crucial two-way experimental condition × self-monitoring interaction also emerged as significant, *F*(1, 203) = 5.41, *p* = .021, 95%C.I. [0.06, 0.72]. Simple slope analysis revealed that both participants with lower levels of self-monitoring (–1SD) and participants with higher levels of self-monitoring (+1SD) reported lower perception of control in the cyber-ostracism condition (vs. cyber-inclusion). However, contrary to our expectations, levels of perceived control did not differ in the cyber-ostracism condition, but in the cyber-inclusion condition. More specifically, participants with higher levels of self-monitoring (+1SD) reported higher levels of control in the cyber-inclusion condition, *b* = 1.51, *SE* = 0.22, 95%CI [1.07, 1.96], *t*(203) = 6.73, *p* < .001, compared to participants with lower levels of self-monitoring (–1SD), *b* = 0.77, *SE* = 0.22, 95%CI [0.33, 1.22], *t*(203) = 3.45, *p* < .001, (see Figure 2). The magnitude of the effect was larger for participants with higher levels of self-monitoring. Crucially, self-monitoring did not moderate any of the other subdimensions of need satisfaction *Fs* < 0.293, *ps* > .589.

## General Discussion

The present study mainly aimed to explore the role of self-monitoring as a key individual variable in moderating the impact of cyber-ostracism on people’s emotions and need satisfaction. In doing so, we employed the Ostracism Online paradigm (Wolf et al., 2015), a research tool resembling a social media platform that allows researchers to manipulate the number of likes that participants receive as an indicator of cyber-ostracism. Our findings showed for the first time that self-monitoring can indeed modulate the impact of cyber-ostracism, extending the wide literature on individual differences in self-monitoring levels. However, results highlighted a different moderating role of self-monitoring on emotions and need satisfaction, confirming previous literature showing that emotions and need satisfaction cannot overlap (Eck & Riva, 2016).

With regard to the emotional reactions, results confirmed our hypothesis: in the cyber-ostracism condition, participants with higher levels of self-monitoring reported higher levels of negative emotions compared to participants with lower levels of self-monitoring. This finding confirms the importance that people with higher levels of self-monitoring give to the social image (Snyder, 1974) and supports the hypothesis that their sensitivity to the likes received by other people may be interpreted as a signal of social acceptance.

Regarding the need for satisfaction, we hypothesized that self-monitoring would moderate the effect of being cyber-ostracized. Our results partially confirmed our hypothesis. In fact, self-monitoring did not moderate the relation between cyber-ostracism and the overall need satisfaction index. This result supports the Temporal Need Threat Model (Williams, 2009), according to which moderation is unlikely to be observed at a reflexive stage than at a reflective one when considering the overall need satisfaction index. However, when exploring results on the sub-components of the need satisfaction, we did find a significant moderating effect of self-monitoring on the perceived sense of control. Hence, our results suggest that self-monitoring might moderate the effect of cyber-ostracism on some dimensions of need satisfaction and not others. Additionally, we did not find differences in perceived control in the cyber-ostracism condition, but in the cyber-inclusion condition. Specifically, included participants with higher levels of self-monitoring reported higher levels of perceived control compared to included participants with lower levels of self-monitoring. In this regard, previous literature highlighted inconsistent results regarding the control dimension in studies that employed the Ostracism Online paradigm. For example, whilst Wolf and colleagues (2015) and Lutz & Schneider (2021) found effects of cyber-ostracism on all the dimensions of need satisfaction, other studies (see e.g., Ren et al., 2021; Schneider et al., 2017; Timeo et al., 2020) did not find effects of cyber-ostracism on the control dimension. Timeo and colleagues (2020) argued that, differently from the Cyberball task, in the Ostracism Online paradigm excluded participants are allowed to act (e.g., liking others’ profiles) while in the Cyberball task ostracized participants cannot throw the ball when they do not receive it. Similarly, Schneider and colleagues (2017), suggested that excluded participants in the Ostracism Online might act to restore their control, e.g., like others’ profiles in an attempt to stimulate their responses, whereas in the Cyberball task, there is no such opportunity. With regard to the present research, we observed a main effect of the task, with cyber-included participants showing higher control than cyber-ostracized participants, but we also observed a main effect of self-monitoring, which was positively associated with the perceived control. Most importantly, in the inclusion condition, participants with higher levels of self-monitoring showed higher levels of control compared to participants with lower levels of self-monitoring. This unexpected result highlights the importance of considering inclusion as a condition that can, per se, lead to outcomes that are not necessarily the opposite of social exclusion. In this regard, using an online ostracism paradigm resembling Facebook, Karlen & Daniels (2011) showed significant differences in the social monitoring skills (i.e., the tendency to use social cues to gather information about the social world) between cyber-ostracized and cyber-included participants. Specifically, only included participants were more likely to misinterpret facial stimuli that showed high-intensity anger. According to the authors, having experienced a social scenario during which they received positive feedback, cyber-included participants were less likely to assume that an intense negative emotion would be directed at them. De Panfilis and colleagues (2015) further showed how social inclusion could be a multi-facet condition. In a study considering patients with borderline personality disorder, they found no differences between the emotions of included and excluded participants after a Cyberball manipulation. Interestingly, negative emotions decreased only within the over-inclusion condition. According to the authors, under some circumstances, social expectations could be satisfied in an over-inclusion rather than in the inclusion situation. With regard to our unexpected result, we reasoned that higher levels of self-monitoring might have elicited an illusionary sense of control in the cyber-inclusion condition, given that high self-monitors may interpret the received ‘likes’ as a confirmation of their ability to control their image and expressive behavior (i.e., receiving ‘likes’ as a result of giving ‘likes’ or receiving appreciation of their self-description), even though all ‘likes’ received were pre-programmed. In other words, it may be possible that, for high self-monitors, the received likes created a condition that satisfied their need to control the contextual cues. However, we can only speculate on the reasons behind this result, given that, to the best of our knowledge, this is the first time that self-monitoring is studied in relation to social inclusion. Indirect support of our reasoning derived from the wide literature about self-monitoring which showing that high self-monitors are motivated to acquire cues for socially desirable behaviors to guide their impression management activity by looking at what others are doing (Jang et al., 2016; Martin et al., 2000; Snyder, 1974). Future research should better address inconsistent results regarding the control dimension and clarify these effects.

From a more empirical and methodological perspective, our findings suggest that the Ostracism Online paradigm is a useful tool to study cyber-ostracism that occurs within social media, due to its high ecological validity. Confirming previous literature on social exclusion and ostracism reactions, our study shows that being liked (or disliked) on a social network impacted the well-being of young adults in terms of emotional reactions (e.g., Buckley et al., 2004; Chow et al., 2008; see Paolini, 2019 for a review) and need satisfaction (e.g., Williams, 2009; Wolf et al., 2015). In fact, similar to previous studies employing Ostracism Online (e.g., Reich et al., 2023; Schneider et al., 2017) cyber-ostracism experimented during the task increased the participants’ negative emotions and affected their basic needs, leading to an overall lowering of their need satisfaction. These findings are particularly relevant nowadays when 1.8 billion people across the world use Facebook. Also, the increment in social network usage observed during the Covid-19 emergency showed that people took refuge on social networks to fight the social isolation caused by the health emergency (Jaspal et al., 2020). However, when social networks become the place of cyber-ostracism, people feel hurt in terms of both emotional reactions and basic need satisfaction with negative consequences for their well-being. At the same time, our results showed how a dispositional factor such as self-monitoring can affect the consequences of the ostracism experienced online given its different moderating role on emotions and control. However, some limitations must also be acknowledged. The most important limitation of the present study regards the effect solely found on the control dimension of need satisfaction when considering self-monitoring as a moderator. We speculated that this result may be due to the theoretical construct of self-monitoring and its nature (e.g., high self-monitors try constantly to adapt their behavior to the contextual request, and within the inclusion condition, they may believe that they are acting adequately to the context requests) but also to the features that characterize Ostracism Online. In particular, differently from the Cyberball task, Ostracism Online allows participants to act, whether they are cyber-ostracized or cyber-included. This aspect of the paradigm may also be responsible for the inconsistent results documented in the literature. Future research should consider these inconsistencies and help disentangle differential results of self-monitoring for emotions and control. On the other hand, we believe that the possibility to act or (believe to) interact with other users is an essential characteristic to consider when studying cyber-ostracism, as social media are based on participation and interaction. Conversely, even though some studies did find moderation at a reflexive stage (see Hartgerink et al., 2015), Williams and Nida (2022) noted that a moderating effect is unlikely to be observed at a reflexive stage when considering the overall need satisfaction index. Further research should investigate the possible moderating role of self-monitoring at a reflective stage. Another limitation of the present research is that it focuses just on likes received as a signal of online ostracism. However, social networks are complex realities that offer different features, and online ostracism can be perpetrated also through other means (e.g., comments). Future studies should consider other cues of online ostracism as well as explore ostracism occurring within different social networks (e.g. Instagram, Twitter, Tik-Tok). Lastly, fake profiles were not pre-tested but simply translated from the original version (Wolf et al., 2015) and adapted to the Italian context. Although other authors have also used our procedure (e.g., Schneider et al., 2017), pretesting fake profiles could represent added value in future research.

## Data Availability and Open science statement

The data underlying the results presented in the experiments are available through the Open Science Framework (https://osf.io/sm2rt/?view_only=5b86f14d2f394ffca5ce163da36c6702).

## Footnotes

GAMLj package in jamovi commonly uses simple contrast coding that compares the reference group to each of the other groups, rather than effect or dummy coding. However, it centers the contrast to 0 (i.e., the average of the sample), so that the other variables’ effects are computed on average.

Main analyses were also run including participants that failed the attention check. The attention check failure did not affect our results. Furthermore, all participants that failed the attention check belonged to the inclusion condition (i.e., they were not able to recall the exact number of likes received, but they recalled receiving more than three likes).
